# No one data source captures all: A nested case-control study of the completeness of maternal death reporting in Banten Province, Indonesia

**DOI:** 10.1371/journal.pone.0232080

**Published:** 2020-05-07

**Authors:** Siti Nurul Qomariyah, Reena Sethi, Yulia Nur Izati, Tika Rianty, Kamaluddin Latief, Ali Zazri, Besral, Massee Bateman, Eskaning Arum Pawestri, Saifuddin Ahmed, Endang L. Achadi

**Affiliations:** 1 Jhpiego Indonesia, Jakarta, Indonesia; 2 Jhpiego, Baltimore, Maryland, United States of America; 3 Center for Family Welfare, Faculty of Public Health, University of Indonesia, Depok, Indonesia; 4 USAID Indonesia, Jakarta, Indonesia; 5 Johns Hopkins University, Bloomberg School of Public Health, Baltimore, Maryland, United States of America; University of Southampton Faculty of Medicine, UNITED KINGDOM

## Abstract

**Background:**

Indonesia’s national health information systems collect data on maternal deaths but the completeness of reporting is questionable, making it difficult to design appropriate interventions. This study examines the completeness of maternal death reporting by the district health office (DHO) system in Banten Province.

**Method:**

We used a nested-control study design to compare data on maternal deaths in 2016 from the DHO reporting system and the MADE-IN/MADE-FOR method in two districts and one municipality in Banten Province, with the aim of identifying and characterizing missed deaths in the DHO reporting system. The capture-recapture method was used to assess the magnitude of underreporting of maternal deaths by both systems.

**Results:**

A total of 169 maternal deaths were reported in the MADE-IN/MADE-FOR study for calendar year 2016 in the three study areas. The DHO system reported 105 maternal deaths for the same period, of which 90 cases were found in both data sources. Capture-recapture analyses suggest that the MADE-IN/MADE-FOR approach identified 92% (95% CI: 87%–95%) of all maternal deaths, while the DHO system captured 57% (95% CI: 50%–64%) of all maternal deaths. Deaths of women who resided in urban areas had four times higher odds (OR 4.3, 95% CI: 1.52–12.3) of being missed by the DHO system compared to deaths among women who lived in rural or remote areas after adjusting for other covariates.

**Conclusion:**

The DHO reporting system missed approximately half of the maternal deaths in the 3 study areas, suggesting that the DHO system is likely to grossly underestimate the maternal mortality ratio. The DHO reporting system needs to be improved to capture and characterize all maternal deaths.

## Introduction

The World Health Organization (WHO) defines a maternal death as “*the death of a woman while pregnant or within 42 days of termination of pregnancy*, *irrespective of the duration and site of the pregnancy*, *from any cause related to or aggravated by the pregnancy or its management*, *but not from accidental or incidental causes* [1].” Over the past two decades, the number of maternal deaths and the maternal mortality ratio (MMR) have declined globally, but in Indonesia the MMR remains high (more than 300 maternal deaths per 100,000 live births) [2,3]. The maternal mortality ratio is one of the key indicators for tracking global Sustainable Development Goal number 3 but its measurement is difficult in settings where the vital registration system is weak, as it is in Indonesia. Local information on the occurrence, timing and causes of maternal death, as well as key contributing factors, is vital to guide the design and implementation of program interventions to reduce maternal mortality in a specific context. However, maternal deaths are rare and accurate information on maternal deaths and underlying causes is often difficult to obtain, in part due to inconsistent definitions and classification and failure to capture all deaths. In addition, many low-resource countries lack robust vital registration systems that capture all maternal deaths [4].

Ideally, data on maternal deaths should come from routine reporting systems to ensure that data are up to date and available at sub-national levels to guide local planning and policymaking [5]. This is especially relevant in the Indonesian setting where decentralization of the health care system requires sub-national district-level decision making. In Indonesia, routine health information systems and vital registration systems often fail to capture accurate data on maternal deaths and therefore are inadequate to guide local maternal health policy, programming and resource allocation [6]. Despite these weaknesses, national- and district-level officials in Indonesia continue to rely on the routine health information system (HIS) to obtain information about maternal deaths.

Information flow in Indonesia’s HIS begins with “bottom-up” reporting from health centers to the district health offices (DHOs), then to provincial health offices, and finally to the national-level Ministry of Health (MOH). Vital events, such as births and deaths, are reported through either the HIS or the government’s civil registration system, which collects data from village offices and sub-district offices. At the health center level, the HIS reporting system incorporates both facility and community data on maternal deaths and mainly relies on village midwives (*bidans*) to report maternal, newborn, and child health-related events (births, deaths) that occur in their catchment areas. When reporting maternal deaths, for example, a village midwife reports maternal deaths that occur in the home and in the health facilities in her catchment area (including both public and private facilities). This reporting system operates in the same way in both rural and urban areas.

The purpose of this study is to provide information on the quality of maternal death reporting to Indonesia’s MOH and local district governments and to make suggestions for ways to improve maternal death reporting. Specifically, the study aimed to measure the completeness of the DHO system’s in reporting maternal deaths and to characterize the maternal death cases that were missed and that were captured by the DHO reporting system, including the factors associated with deaths that were missed by the DHO reporting system.

The research presented in this paper is part of a larger study undertaken in three districts in Banten Province, Indonesia (the Banten II Study) to measure maternal mortality and the determinants of maternal deaths. Banten is one of the MOH’s focus provinces because of its high number of maternal deaths. The results of this study may be relevant for other countries facing similar challenges in reliably capturing maternal deaths in their routine HIS.

## Methods

### Study design

This paper used a subset of data on maternal deaths in 2016 from a nested case-control study in two districts and one municipality in Banten Province (the Banten II Study), the objective of which was to measure maternal mortality using the Maternal Death from Informants/Maternal Death Follow-On Review (MADE-IN/MADE-FOR) method [7]. The MADE-IN/MADE-FOR method was developed and first used approximately ten years ago and was found to be sensitive in capturing all maternal death in a specific location [8]. Cases were defined as maternal deaths that were not captured by the DHO reporting system (missed cases), and controls were defined as deaths that were captured by the DHO reporting system (reported cases). The Banten II Study collected information about characteristics of both missed cases and reported cases using the MADE-IN/MADE-FOR method.

### Data collection methods

Information on maternal deaths in 2016 in the three study areas was collected from two sources, the routine HIS and the two-phased MADE-IN/MADE-FOR method. During the first MADE-IN phase, interviewers asked two types of village informants, the heads of neighborhood units (*RTs*) and health volunteers (*kaders*) to identify all deaths of women of reproductive age in 2016, including information on each woman’s pregnancy/delivery/post-partum status when she died. For the heads of neighborhood unit, those involved in the reporting process were: 1) all those in urban villages; 2) in each subdistrict, one rural village was randomly selected and all head of neighborhood units from the village were involved. For the health volunteers: one health volunteer (the most active one, based on the health center midwife’s judgment) from each health integrated post was selected. All health integrated posts in the districts were represented by one health volunteer each. All informants were invited to gather for separate meetings (i.e. one meeting for health volunteers and one for the heads of neighborhood units) in each sub-district. They were each provided with hard-copy of the reporting form which was attached to the invitation letter to attend the meeting. During the meeting, their understanding on the content of the form was checked to ensure that they could fill in the form correctly. They were not paid for filling in the form but were provided with a small transportation fee of $3.5US each). During the MADE-FOR phase, interviewers visited relatives of the women identified from the MADE-IN phase as having likely suffered a pregnancy-related death; they confirmed that the deaths were indeed pregnancy related and gathered additional information about the cause of death [8].The MADE-IN/MADE-FOR method was first applied in Banten in 2005–2006 to measure maternal deaths. The same method and survey instruments for collecting information on maternal deaths were used for the Banten II Study; however, the questionnaire for interviewing the family of the deceased was revised slightly to align with WHO’s latest version of the verbal autopsy form [9].

Data on maternal deaths occurring in 2016 were also collected from the three DHO reporting systems. These sources included verbal autopsy forms completed by health centers in the three districts as well as the lists of maternal deaths compiled by DHO staff, which included deaths that occurred in hospitals in the three districts. The data at each health center come from village midwife reports. Village midwives record maternal death cases in their catchment areas using hard copy verbal autopsy forms which include some information on the characteristics of maternal deaths.

### Study site and period

This study used data from two districts and one municipality of Banten Province, Indonesia: Serang District, Serang Municipality, and Pandeglang District. Although the larger Banten II Study collected data on maternal deaths that occurred between July 1, 2015 and June 30, 2017, the case-control analysis in this study was limited to maternal deaths occurring in the year 2016. This was because information on maternal deaths in the DHO system specified only the year of death and did not provide information on the exact date of death. Thus, it was necessary to limit the comparative analysis of DHO reporting and Banten II study data on maternal deaths to deaths that occurred in 2016.

### Data management and quality assurance

To assure the quality of the data collected through the MADE-IN/MADE-FOR method, all interview forms were rechecked by the data collectors to ensure that answers were clear and complete. Data collectors who mostly had Bachelor degrees in public health, midwifery or other related areas were trained on research ethics, the study protocol and tools. The completed questionnaires were rechecked again by the field supervisor before data were entered into an Epi- Info^™^ (Atlanta, GA, USA) database. The data abstracted from the DHO recording system were copied into an MS Excel spreadsheet.

### Data analysis methods

Maternal deaths identified in the DHO routine reporting system were matched with deaths identified through the MADE-IN/MADE-FOR method. The criteria used for matching included the woman’s name, her husband’s name, her address (sub-district, village, etc.), date of death, and pregnancy status at the time of death. Matching was done by listing the cases in MS Excel and sorting by the criteria.

To measure the completeness of the DHO routine reporting system, the capture-recapture technique was used to estimate the total number of deaths, including those missed by both the MADE-IN/MADE-FOR method and the DHO routine reporting system. The capture-recapture method was originally used in ecological studies, primarily for measuring animal abundance [10]. In public health, it is used to determine the size of difficult-to-identify populations [11]. This method involves sampling (capturing) and identifying individuals from a population; resampling (recapture) the population to see what fraction of individuals in the second sample were identified in the first sample; and then using this to estimate the total population: (N1 x N2)/M = T (where T = total population, N1 = number of cases found in the first sample, N2 = number of cases found in the second sample, and M = number of cases found in both samples) [10, 12, 13]. In the MADE-IN/MADE FOR method, the capture and recapture process used the two informant networks described above. N1 is number of cases captured by the health volunteers, N2 is number of cases captured by heads of neighborhood units and M is number of cases captured by both networks.

There were four critical assumptions made in the simple capture-recapture analysis employed here: 1) the population to be estimated is fixed; 2) individuals from both sources can be matched; 3) capture in the second sample is independent of capture in the first sample; and 4) the probability of capture does not differ between individuals.

Descriptive analyses were performed to describe cases missed by the DHO routine reporting system. Bivariate and multivariable analyses were also performed using logistic regression models, adjusting for clustering at the village level. The covariates used in the models include the characteristics of the women (age at death, wealth quartile at death- as measured by questions related to the ownership of selected household goods, district, area of residence at death, and their health insurance schemes), main cause of death, women’s history of antenatal care (ANC) visits (e.g., frequency and the timing of their last ANC visit during their last pregnancy), place of delivery, time of death, and place of death. Significance was set at a p-value of less than 0.05.

### Ethical approval

Ethical approval for the conduct of human subject research was obtained from the Faculty of Public Health, University of Indonesia (No. 450/ UN2.F10/PPM.00.02/2017) and the Johns Hopkins Bloomberg School of Public Health’s Institutional Review Board (No. 7964). Permission was also obtained from the district governments, the DHOs, and the health centers in each study district. Written informed consent was obtained from families of the deceased for the interview/verbal autopsy.

## Results

**Fig 1:**
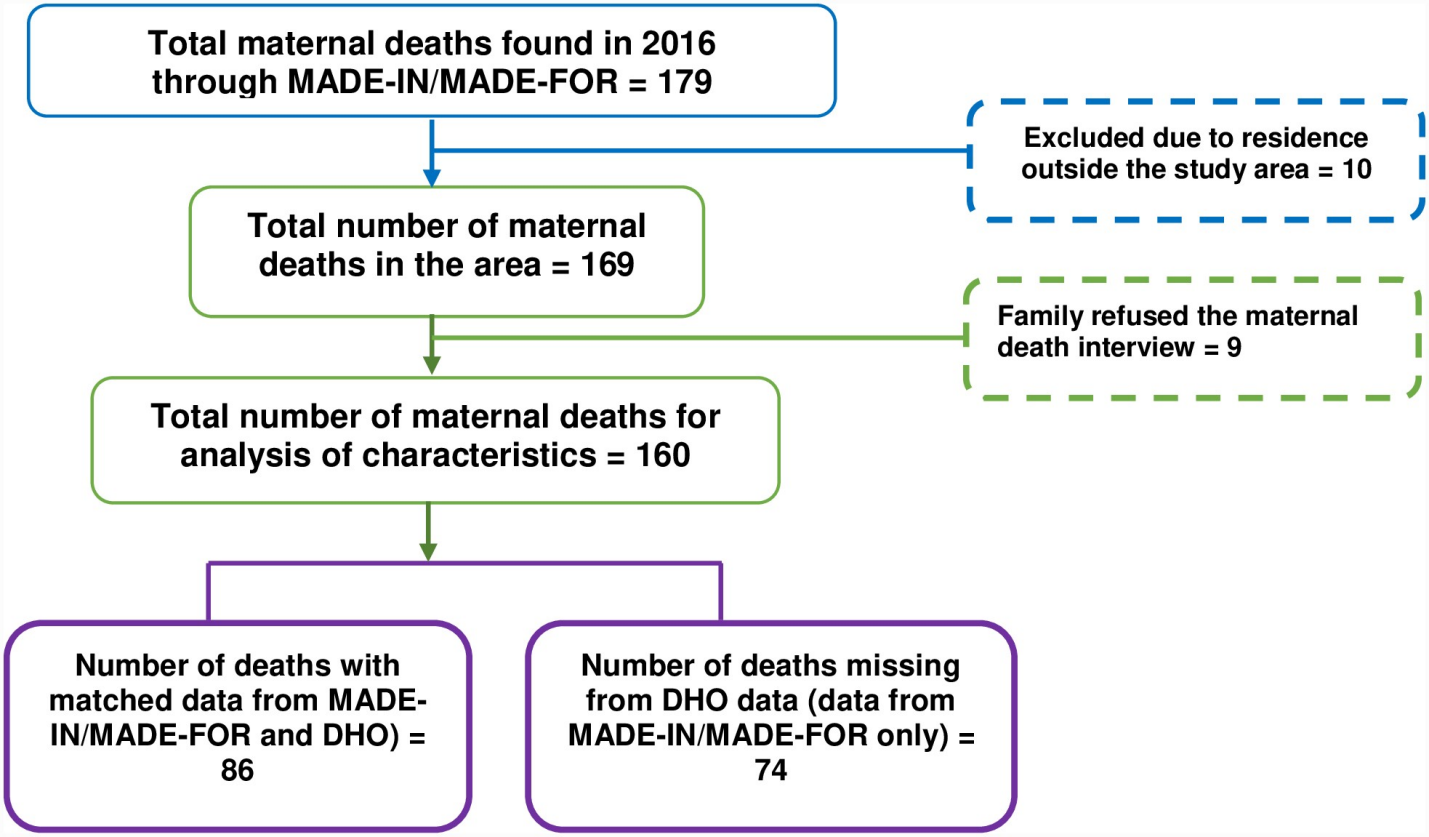
Case identification.

The Banten II MADE-IN/MADE-FOR study reported 341 maternal deaths in the three study districts/municipality between July 2015 and June 2017. Of these, 179 were reported to have occurred in 2016, but 10 of these deaths were excluded from the analysis because the women lived outside the study area. Of the 169 eligible maternal deaths in 2016, only 160 had completed interviews with families of the deceased women (MADE-FOR). The capture-recapture analysis (compared with the DHO data) used the total 169 deaths, whereas the logistic regression analysis used the 160 deaths with complete interview data. Of these 160 deaths, 86 were matched (included in DHO routine reporting) and 74 cases were not matched with the DHO (missed by the DHO routine reporting) (Fig 1).

The DHO reporting system recorded 105 maternal deaths in 2016, while the MADE-IN/MADE-FOR method identified 169 maternal deaths. Nine cases did not have a complete interview because of refusal from the family, leaving 160 cases for this case control analysis (86 maternal death cases found by both MADE-IN/MADE-FOR and the DHO and 74 maternal death cases found only by MADE-IN/MADE-FOR). For the purposes of capture recapture calculation, maternal death cases from the two data sources were matched (see Table 1 below). Using the total 169 cases found by MADE-IN/MADE-FOR, 90 cases were found in both data sources, 79 cases missed by DHO data sources but found by the MADE-IN/MADE-FOR method and 15 cases were missed by the MADE-IN/MADE-FOR method but recorded by the DHO system. Of the 184 total maternal deaths identified by either or both sources, the MADE-IN/MADE-FOR method identified 92% (95% CI: 87%-95%), while the DHO system captured 57% (95% CI: 50%-64%). Neither data source captured all maternal deaths.

**Table 1:** Total number of maternal deaths in 2016 in the three study districts based on the two data sources and capture-recapture analysis.

		MADE-IN/MADE-FOR Method		
		Reported	Not reported	Total
**DHO data set**	Reported	90	15	105
	Not reported	79	13	92
	**Total**	**169**	**28**	**197**

The capture-recapture technique was then applied to estimate the total number of maternal deaths, including those that were missed by both data sources. Using the capture-recapture formula (N1 X N2/M, with N1 [number of cases captured by the first network] = 105, N2 [number of cases captured by the second network] = 69, and M [number of cases captured by both networks] = 90), the estimated total number of cases was 197; thus an estimated 13 maternal deaths were missed by both data sources. Based on the total number of cases estimated through the capture-recapture approach, the completeness of the DHO’s reporting of maternal deaths in 2016 was 53% while the completeness of maternal deaths reporting by the MADE-IN/MADE-FOR method was 86% (Table 1).

We further explored the 15 cases missed by the MADE-IN/MADE-FOR method. Of these, five cases were in fact found by the MADE-IN/MADE-FOR method but were not included in the study’s final dataset because village informants said they were ineligible for various reasons: one case was ineligible due to the date of death; two cases were from districts outside the study area; the health volunteer (one of the village informants) was not certain if one case was a maternal death; and the village informant said one case was a non-maternal death although the DHO reported it as a maternal death. Of the remaining 10 missed cases, three could not be matched because the woman’s name was not included in the DHO data set; two of these three cases also lacked information on the date of death and address of the woman. The other seven cases did not exist in the MADE-IN/MADE-FOR data set at all.

### Characteristics of the maternal death cases

Table 2 shows the characteristics of maternal deaths in the three study districts in 2016 that were missed and that were captured by the DHO reporting system. There were statistically significant differences between missed and captured deaths with respect to the individual district and with respect to the urban versus rural residence of the women who died. Among the missed maternal deaths, most women were between 20–35 years of age when they died (76.7%). Most deaths occurred in rural/remote areas (72.5% overall), but the DHO information system captured nearly 85% of those cases, implying that more cases from urban area were missed by the DHO reporting system. By district, a higher proportion of missed cases were found in Pandeglang compared to the other two districts (37.8% compared to 32.4% and 29.7% for Serang City and Serang District, respectively) even though Serang District accounted for nearly half of the death cases. In contrast, for cases that were not missed, more than half (55.8%) were in Serang District and lower proportions were in Pandeglang and Serang City (32.6% and 11.6% retrospectively). There were no significant differences by insurance scheme or wealth quartile.

**Table 2:** Socio-economic characteristics of maternal deaths in three districts of Banten Province in 2016, by whether they were captured by the DHO reporting system.

Characteristics of maternal deaths	Missed by DHO (%)	Captured by DHO (%)	(*p* value)	Total (%)
**Age**	**n = 73 (%)**	**n = 86 (%)**		**n = 159**
<20	8 (11.0%)	10 (11.6%)	*ref*	18 (11.3%)
20–35	56 (76.7%)	49 (57.0%)	0.453	105 (66.0%)
>35	9 (12.3%)	27 (31.4%)	0.108	36 (22.6%)
**Wealth quartile**	**n = 73**	**n = 86**		**n = 159**
Q1	21 (28.8%)	32 (37.2%)	*ref*	53 (33.3%)
Q2	27 (37%)	19 (22.1%)	0.022	46 (28.9%)
Q3	12 (16.4%)	18 (20.9%)	0.965	30 (18.9%)
Q4 (wealthiest)	13 (17.8%)	17 (19.8%)	0.765	30 (18.9%)
**District**	**n = 74**	**n = 86**		**n = 160**
Serang District	22 (29.7%)	48 (55.8%)	*ref*	70 (43.8%)
Serang City	24 (32.4%)	10 (11.6%)	**< .001**	34 (21.3%)
Pandeglang	28 (37.8%)	28 (32.6%)	**0.003**	56 (35.0%)
**Residence**	**n = 74**	**n = 86**		**n = 160**
Rural/remote	43 (58.1%)	73 (84.9%)		116 (72.5%)
Urban	31 (41.9%)	13 (15.1%)	**<0.001**	44 (27.5%)
**Insurance schemes**	**n = 74**	**n = 86**		**n = 160**
Other insurance	18 (24.3%)	21 (24.4%)	*ref*	39 (24.4%)
No insurance	35 (47.3%)	38 (44.2%)	0.877	73 (45.6%)
Subsidized insurance	21 (28.4%)	27 (31.4%)	0.835	48 (30.0%)

A quarter of the maternal deaths missed by the DHO reporting system were attributed to indirect causes (25.7%) based on verbal autopsy. According to WHO, indirect causes of maternal deaths include previously existing diseases or diseases that developed during pregnancy that are not due to direct obstetric causes but are aggravated by physiologic effects of pregnancy [1]. This was significantly higher than for cases captured by DHO (13.9%). More than one third (34.7%) of cases missed by the DHO had their last ANC visit before the third trimester or had no ANC. More than half of the missed cases (55.4%) died more than 24 hours postpartum and more than one-quarter (27%) died during pregnancy (antepartum). In addition, almost 40% of the missed death cases delivered at home (Table 3).

**Table 3:** Maternity care and pregnancy-related characteristics of the maternal deaths in three districts of Banten Province in 2016, by whether they were captured by the DHO reporting system.

Characteristics of maternal deaths	Missed by DHO (%)	Captured by DHO (%)	(*p* value)	Total (%)
**Cause of death**	**n = 74**	**n = 86**		**n = 160**
Direct	55 (74.3)	74 (86.1)		129 (80.6%)
Indirect	19 (25.7)	12 (13.9)	0.070	31 (19.4%)
**ANC**				
**Frequency of ANC visits**	**n = 67**	**n = 83**		**n = 150**
4+ visits	49 (73.3%)	66 (79.5%)		115 (76.7%)
<4 visits	18 (26.9%)	17 (20.5%)	0.409	35 (23.3%)
***First ANC***	**n = 68**	**n = 81**		**n = 149**
1^st^ trimester	46 (67.7%)	62 (76.5%)		108 (72.5%)
Not in 1^st^ trimester or no ANC	22 (32.4%)	19 (23.5%)	0.249	41 (27.5%)
***Last ANC***	**n = 72**	**n = 83**		**n = 155**
Before 3^rd^ trimester or no ANC	25 (34.7%)	12 (14.5%)		37 (23.9%)
3^rd^ trimester	47 (65.3%)	71 (85.5%)	**< .001**	118 (76.1%)
**Place of delivery end**	**n = 50**	**n = 69**		**n = 119**
Facility	30 (60.0%)	48 (69.6%)		78 (65.5%)
Home	20 (40.0%)	21 (30.4%)	0.173	41 (34.5%)
**Time of death**	**n = 74**	**n = 86**		**n = 160**
Intrapartum	6 (8.1%)	12 (13.9%)	*ref*	18 (11.2%)
During pregnancy	20 (27.0%)	16 (18.6%)	0.067	36 (22.5%)
Immediate postpartum (up to 24 hours after delivery)	7 (9.5%)	30 (34.9%)	0.092	37 (23.3%)
Delayed postpartum (24+ hours to 42 days after delivery)	41 (55.4%)	28 (32.6%)	0.051	69 (43.1%)
**Place of death**	**n = 74**	**n = 86**		**n = 160**
Facility	45 (60.8%)	67 (77.9%)		112 (70.0%)
Home	29 (39.2%)	19 (22.1%)	**0.033**	48 (30.0%)

### Predictors of maternal death cases missed by the DHO reporting system

Women’s place of residence (rural/remote vs. urban) was related to the odds of being missed by the DHO reporting system. Maternal deaths of women who resided in urban areas were four times more likely (adjusted odds ratio [aOR] 4.3, p value = 0.006) to be missed by the DHO system compared with maternal deaths of women who lived in rural or remote areas. Women who had their last ANC visit before the third trimester or who did not have any ANC visits were nearly three times more likely (aOR 2.9, p value = 0.019) to be missed by the DHO system. The odds of a maternal death during delivery being missed was 70% less than a maternal death during pregnancy being missed (aOR 0.30, p value = 0.035). For women who died at home, the odds of being missed were 2.16 (p value < 0.001) compared with women who died in health facilities. There was no relationship between the frequency of ANC visits with the odds of being missed by the DHO reporting system (aOR 0.73, p value = 0.617) (Table 4).

**Table 4:** Results of multivariable logistic regression analysis of variables associated with maternal deaths missed by the DHO reporting system in three districts of Banten Province in 2016.

Characteristics	OR (95% CI)	*p* value	Adjusted OR (95%) CI	*p* value
Residence in urban area	4.04 (2.11–7.77)	**< .001**	4.31 (1.52–12.3)	**0.006**
Last ANC visit before third trimester or no ANC visit	3.14 (1.94–5.10)	**< .001**	2.93 (1.19–7.22)	**0.019**
Less than 4 ANC visits	1.43 (.614–3.31)	0.409	0.73 (0.39–1.37)	0.329
Timing of death (ref: during pregnancy)				
Up to 24 hours after delivery	0.467 (.192–1.13)	0.092	0.30 (0.10–0.92)	**0.035**
24+ hours after delivery	2.92 (.996–8.61)	0.051	0.95 (.584–1.53)	0.821
Death at home	2.27 (1.07–4.83)	0.033	2.16 (1.52–3.08)	**< .001**

## Discussion

The DHO reporting system missed approximately half of the maternal deaths that occurred in the three study areas in Banten Province in 2016, meaning that the exclusive use of maternal death data from the DHO reporting system will result in a substantial underestimation of the maternal mortality ratio in these 3 areas. Using the DHO reporting system may therefore also lead to incorrect assumptions about the characteristics of maternal deaths, including place of death, woman’s place of residence, and other key factors.

Our study found that the DHO reporting system missed more maternal deaths of women in urban areas than in rural areas. While the DHO reporting system is ostensibly the same in rural and urban areas, urban and rural areas face different reporting implementation challenges, particularly with respect to community-level reporting of maternal deaths. In the Indonesia reporting system, reporting events (including maternal death) mostly relies on the report from village midwives. Most rural villages in Indonesia have one village midwife, so the reporting for rural areas is more structured. Some urban areas have no midwife assigned and reporting of deaths depends on many private facilities which exist there. In addition, the population in urban areas is mostly higher than in rural villages. This may result in the inability of the village midwife to adequately cover an entire village in more population-dense urban areas. There is also a greater concentration of private clinics in urban areas, which may be another factor that contributes to village midwives being unable to identify all cases. This is similar to findings from a study in Iran [14], where the basis for information registration and collection in urban areas is the household record but the lack of a logical relationship between providers results in insufficient information on clients.

Several studies have found an association between the number of ANC visits and institutional delivery [15,16]. We assumed that having more ANC contacts with health care providers would positively affect the likelihood of a woman’s pregnancy-related death being recorded due to an increased number of contacts with the formal health care system. However, our study found that the number of ANC visits a woman had was not related to the odds of her death being captured by the DHO reporting system. This finding implies a need to increase awareness among health care providers of the importance of follow-up with their ANC clients to retain women in ANC care and to obtain information about their pregnancy outcomes.

Globally, most maternal deaths occur in the intrapartum and early postnatal periods [17]; the highest proportion of maternal deaths in this study captured by the DHO reporting system occurred during the early postpartum period up to 24 hours (34.9%) and the delayed postpartum period after 24 hours (32.6%), with 13.9% occurring during the intrapartum period and 18.6% of maternal deaths occurring during the antenatal period. A significant proportion of deaths that occurred in the antepartum and late postpartum period were missed by the DHO reporting system. Based on the policy of Indonesia’s MOH, all postpartum women should have three postpartum visits, which consist of a first visit that occurs between 6 hours and 3 days after delivery, a second visit that occurs between 4 and 28 days postpartum, and a third visit that occurs between 29 and 42 days after delivery. In Indonesia, the latest national-level figures of third postpartum visit coverage, based on the 2015 Health Profile, is quite high, as much as 87% [18]. One possible explanation for the high proportion of unreported maternal deaths in the late postpartum period is that last postpartum visits are often conducted before the end of the period (42 days postpartum).

Compared to women who died during pregnancy, those who died during delivery were three times less likely to be missed by the DHO reporting system. This is reasonable because the high coverage of facility deliveries (63% based on Indonesia Demographic and Health Survey 2012) [2] might lead to high completeness of death reporting during this period.

This study also found that women who died at home were about two times more likely to be missed by the DHO reporting system. This is understandable as those who died at home might not have contacts with health providers, so the events would not be captured by the DHO routine reporting system.

Until now, and like many other low- to middle-income countries, Indonesia has relied on surveys (Indonesia Demographic and Health Surveys), population censuses, and intercensal surveys for maternal mortality estimates. However, the national-level MMR estimates generated by surveys are only available every 5 years. Ideally, routine HIS and vital registration systems can provide an alternative to more local and real-time sources of information on maternal deaths; however, the quality of maternal death reporting in these systems is low. The findings in our study are similar to those of several other studies. For example, a study in Mozambique showed that health institutions failed to register 86% of maternal deaths [19]. Another study in Morocco found underreporting of maternal deaths by 58% in the Gharb Chrarda Bni Hssen Region in 2014 [20]. Using a reproductive age mortality approach (RAMOS), which captures maternal deaths using all existing data sources on deaths of women of reproductive age, studies in Ghana and Malawi [21] showed that official sources underreported maternal deaths by 44% and 43%, respectively. Another study in Taiwan [22] found underreporting of maternal deaths by as much as 65% in official published maternal mortality data.

Our study also reveals the need to strengthen community-based reporting, as there are many women who died at home who were missed by the system. Strengthening facility-based reporting so that village midwives can capture women who died at home will also be crucial. An important consideration in implementing these recommendations is to match data captured from both the community-based and facility-based reporting systems so that the total number of cases can be counted and duplicates can be found and deleted.

The government should target the overall improvement of the civil registration and vital statistics (CRVS) systems, as these are used not only for recording and reporting purpose, but also for meeting the government’s obligation to ensure the right of the people to be counted, as suggested by the United Nations [23]. Improvements in the CRVS system itself might not automatically improve reporting on maternal deaths as most systems, including Indonesia system do not collect information on the pregnancy status of women of reproductive age who die. But, this can be solved by adding a pregnancy status checkbox to the death certificates as is done in many states in the United States, which has likely improved the identification of pregnancy-related deaths [24].

Until there can be complete recording and reporting by the CRVS systems, the routine community-based reporting system in Indonesia can be improved by incorporating periodic surveillance using the MADE-IN/MADE-FOR method, which uses two local informant networks that already exist. This method can be used in other countries with similar measurement challenges to Indonesia, as long as there are local informant networks that are knowledgeable about the vital events that occur in their communities. Implementing the method will ensure the possibility of having MMR estimates available at sub-national level, which will be a crucial source of information for the development of localized policies and strategies at a decentralized level. In addition, intermittent implementation of the method, every 3 years for example, might also be used to evaluate the completeness of the routine information system in reporting maternal deaths. This is very important as studies on maternal mortality measurement continuously prove that there is no one source that captures all maternal deaths [25,26].

The results of this study were presented to policymakers in each of the two districts and one municipality included in the analysis. Despite the discrepancy between the information on maternal deaths provided by the routine DHO reporting system and the MADE-IN/MADE-FOR method, policymakers indicated that they plan to continue to use routine HIS data so that their numbers will be comparable with reports from other districts using the same routine HIS data sources. There is an important opportunity for the national government and provincial and district health managers to leverage the learning from this study to strengthen the accuracy of maternal death reporting in the routine health information system to inform local programming to eliminate preventable maternal mortality. Some of the actions that could be taken by the Indonesian government in improving the maternal death reporting are as follows: 1) ensuring all hospitals record and report maternal deaths to DHO, 2) ensuring private facilities are involved in the reporting system, 3) ensuring the complete reporting of maternal deaths by firstly identifying all deaths of women of reproductive age, then identifying the pregnancy status at the time of death.

### Limitations

The data used for this study are limited to maternal deaths that occurred in 2016 in two districts and one municipality, so the total number of deaths is small and may not be generalizable to other time periods or areas of the country. The data about the characteristics of women who died, including data on cause of death, number and timing of ANC visits, were collected through the MADE-FOR visit and do not exist in the DHO routine reporting system. Even with these limitations, this study is valuable because it provides information that did not exist and can be used by the Indonesian MOH to improve the completeness of maternal death reporting at district and national level.

## Conclusion

The MADE-IN/MADE-FOR method identified 61% more maternal deaths as compared with the DHO reporting system. Based on our findings, efforts to improve the completeness of reporting of maternal deaths in the DHO reporting system should include postnatal visits on the last day of the postpartum period (42 days after delivery) to ensure that women who died during that period are not missed. Midwives should be encouraged to track women who are in their 3^rd^ trimester of pregnancy who have not come in for antenatal care. To adequately identify all maternal deaths of women residing in urban areas, the community-based reporting system that is based on reporting by the village midwives may need to be revisited and strengthened. One village midwife may also not be able to cover the entire population within an urban village so catchment areas covered by these midwives should be reviewed. Ensuring private clinics’/facilities’ participation in the reporting system is also crucial for completeness.
